# Economic Problems and Mental Disorders in Japanese Pregnant Women

**DOI:** 10.14740/jocmr2049w

**Published:** 2015-03-01

**Authors:** Shunji Suzuki

**Affiliations:** Department of Obstetrics and Gynecology, Japanese Red Cross Katsushika Maternity Hospital, 5-11-12 Tateishi, Katsushika-ku, Tokyo 124-0012, Japan. Email: czg83542@mopera.ne.jp

## To the Editor

Maternal mental disorders are associated with adverse psychological outcomes in children in countries of low and middle income [1, 2]. Although we believe that Japan is not a poor country, there are some morbid pregnant women who are impoverished economically. Between 2009 and 2013, for example, at our hospital 226 (3.7%) of 6,119 pregnant women could not give birth without financial support from the hospitalization assistance policy (HAP) system by the Japanese Child Welfare Government. The HAP system aids their delivery costs. The main objectives of the HAP system are to help pregnant women who: 1) receive the livelihood protection because they are unable to maintain minimum living standards because of poverty, 2) live in households exempt from the residence tax, and 3) live in households in which the income tax is less than ¥8,400 (about 80 US dollars) per year. In the women without economic problems, 84 women (1.4%) were diagnosed as having mental disorders such as schizophrenia and/or depression requiring medications before or during pregnancy; however, 16 women (7.1%, P < 0.01 by Chi-square test) were diagnosed as having the mental disorders in women under the HAP system. During the pregnancy, in the 84 women of normal economic state, 19 women (22.6%, P < 0.01 by Chi-square test) performed self-interruption of medications and 15 women (17.9%) had the worsening of symptoms; however, in the 16 pregnant women with mental disorders under the HAP system, 10 women (62.5%) performed self-interruption of medications and seven women (43.8%, P = 0.02 by Chi-square test) had the worsening of symptoms.

Women with mental disorders sometimes perform self-interruption of medications during pregnancy, and then the self-interruption of medications sometimes deteriorates their state of mental disorders. They seemed to misunderstand that the medication leads to the adverse outcomes of pregnancy. In addition, the pregnant women impoverished economically may be liable to be as follows: 1) they have often received low education leading to their misunderstanding about the influence of medications on their pregnancies because their parents had been also poor; 2) there may be large time and/or economic burdens in their visiting of psychiatric and obstetrics; and 3) the stress of pregnancy may be easy to hunt down their psychological status because there is no room in their life. These may become negative chains in their lives and these may lead to the poverty and adverse psychological outcomes of their children. The Japanese HAP system aids their delivery costs only. Therefore, in Japan the proper education and sufficient financial support are needed for pregnant women who are impoverished economically to prevent the deterioration of their mental status during pregnancy.
